# Posttraumatic stress disorder–related anhedonia as a predictor of psychosocial functional impairment among United States veterans

**DOI:** 10.1002/jts.22832

**Published:** 2022-04-11

**Authors:** Casey L. May, Blair E. Wisco, Victor A. Fox, Brian P. Marx, Terence M. Keane

**Affiliations:** ^1^ Department of Psychology University of North Carolina at Greensboro Greensboro North Carolina USA; ^2^ National Center for PTSD VA Boston Healthcare System Boston Massachusetts USA; ^3^ Department of Psychiatry Boston University School of Medicine Boston Massachusetts USA

## Abstract

Prior research suggests that anhedonia symptoms related to posttraumatic stress disorder (PTSD; i.e., diminished interest, detachment from others, and difficulty experiencing positive emotions) are consistently associated with a higher degree of impairment in psychosocial functioning beyond that associated with other PTSD symptoms. Unfortunately, much of this research has used cross‐sectional study designs; relied upon outdated *DSM* diagnostic criteria; and failed to control for potentially confounding variables, such as the presence of co‐occurring depression. This study used data from Waves 2 and 4 (*n* = 1,649) of the Veterans’ After‐Discharge Longitudinal Registry (Project VALOR), a longitudinal dataset of U.S. Army and Marine veterans. As measured using the Inventory of Psychosocial Functioning, Wave 4 psychosocial functioning was regressed on seven PTSD symptom factors at Wave 2 (i.e., intrusions, avoidance, negative affect, anhedonia, externalizing behaviors, anxious arousal, and dysphoric arousal) and potential Wave 2 confounds. The Anhedonia factor, β = .123, most strongly predicted later psychosocial functional impairment beyond the impact of other PTSD symptom factors, βs = −.076–.046. Clinical implications of these findings are also discussed.

Posttraumatic stress disorder (PTSD) affects approximately 8% of all United States veterans (Wisco et al., 2016) and is associated with substantial psychiatric comorbidity (Fairbank et al., 2001) and suicidality (Schuman et al., 2019). As currently constituted in the fifth edition of the *Diagnostic and Statistical Manual for Mental Disorders* (*DSM‐5*; American Psychiatric Association [APA], 2013), PTSD is characterized by 20 symptoms parceled into several symptom clusters. Prior research has investigated the associations between PTSD symptom clusters and specific outcomes of interest, such as psychosocial functioning. A systematic review of the findings from 24 studies on the association between PTSD symptom clusters and functional impairment among U.S. combat veterans (Schuman et al., 2019) revealed that symptoms depicting emotional numbing were more strongly related to impairment in psychosocial functioning compared to other PTSD symptoms (e.g., intrusions, avoidance). Emotional numbing and anhedonia are terms often used to reflect dampened appetitive functioning (Kashdan et al., 2006; Pizzagalli, 2014). In prior PTSD research, this construct has been generally described as the cumulative severity of three specific PTSD symptoms, as defined in the fourth edition of the *DSM* (*DSM‐IV*; APA, 1994): “markedly diminished interest or participation in significant activities” (Criterion C4), the “feeling of detachment or estrangement from others” (Criterion C5), and a “restricted range of affect” (Criterion C6; Kashdan et al., 2006; Litz 2003; Litz & Gray, 2002). The separation of these three symptoms from the remaining diagnostic criteria distinguishes the dampened appetitive state portrayed by emotional numbing as opposed to the other cluster symptoms, which more often describe cognition (e.g., negative beliefs about one's self, others, and the world; distorted self‐ and other‐blame; an inability to remember the trauma event).

Importantly, the field's understanding of the association between emotional numbing and psychosocial functioning is confounded by how the construct has been operationalized in the literature. Some prior research has defined “emotional numbing” as either the entire *DSM‐IV* PTSD Criterion C (Campbell & Renshaw, 2013; Riggs et al., 1998; Ross et al., 2018; Shea et al., 2010) or as all Criterion C symptoms except the two symptoms related to avoidance (Allen et al., 2018; Cook et al., 2004; Harder et al., 2011; Nunnink et al., 2010; Ruscio et al., 2002; Schnurr & Lunney, 2011; Sippel et al., 2018). As a result, most prior studies of PTSD‐related emotional numbing have defined the construct more broadly than previously prescribed. Only one study of which we are aware has separated the three specific *DSM‐IV* emotional numbing symptoms from the remaining symptoms in Criterion C, wherein Nunnink et al. (2010) found emotional numbing symptoms to be the strongest predictor of impaired sexual functioning. In addition, the PTSD diagnostic criteria were revised for *DSM‐5*. Most relevant here are the revisions that separated the avoidance and numbing symptoms into two separate symptom clusters and the rewording of the *DSM‐IV* (text revision; *DSM‐IV‐TR*; APA, 2000) symptom “restricted range of affect” (pp. 468) to reflect “dampened positive emotions (e.g., love, satisfaction, happiness)” in *DSM‐5* (APA, 2013; Armour et al., 2015). To date, only one prior study has used *DSM‐5* PTSD criteria to examine these associations, yet the authors did not separate the emotional numbing symptoms from the rest of the *DSM‐5* Criterion D (i.., negative alterations in cognition and mood) symptoms, thus diluting the emotional numbing construct (Ross et al., 2018).

In addition, prior studies often have neglected to account for the role of depression in their analyses (e.g., Allen et al., 2018; Campbell & Renshaw, 2013; Cook et al., 2004; Nunnink et al., 2010; Riggs et al., 1998; Sippel et al., 2018). Dampened emotional responding overlaps between depression and PTSD yet can still be distinguished. For example, PTSD‐related anhedonia includes social detachment or estrangement, a symptom that is not included in the *DSM‐5* diagnostic criteria for depression (APA, 2013; Kashdan et al., 2006), and PTSD symptoms must have occurred following exposure to a traumatic event (i.e., *DSM‐5* Criterion A; APA, 2013). Given that both disorders frequently co‐occur (see Rytwinski et al., 2013, for a meta‐analysis), controlling for the potentially confounding effects of depressive symptoms helps to clarify the independent contributions of each disorder on deficits in functioning. In addition, most prior studies on the association between PTSD‐related emotional numbing and psychosocial functioning have relied heavily on cross‐sectional research designs (e.g., Harder et al., 2011; Nunnink et al., 2010; Riggs et al., 1998; Ross et al., 2018; Ruscio et al., 2002; Schnurr & Lunney, 2011; Sippel et al., 2018; Shea et al., 2010). Unfortunately, cross‐sectional data cannot provide important information about directionality or change over time.

In this study, we sought to improve understanding of the impact of PTSD‐related emotional numbing on functional impairment over and above the contributions of other PTSD symptoms, after controlling for the presence of depressive symptoms, using *DSM‐5* symptom criteria data collected at multiple time points from a sample of U.S. Army and Marine veterans. We defined emotional numbing as the cumulative severity of three symptoms (i.e., diminished interest or participation in significant activities, detachment or estrangement from others, and the inability to experience positive emotions) to examine the unique effect of this construct on functional impairment. We hypothesized that the results would support prior findings (i.e., numbing symptoms would contribute to functional impairment beyond the effects of other PTSD symptoms) and extend the work of other scholars by addressing important limitations of prior research. Specifically, we examined the longitudinal effect of *DSM‐5* emotional numbing symptoms on psychosocial functioning, while controlling for all other PTSD symptom clusters and important covariates, such as major depressive disorder.

## METHOD

### Participants

Data for this study were drawn from participants who completed Wave 2 (*n* = 1,649) of the Veterans’ After‐Discharge Longitudinal Registry (Project VALOR; Rosen et al., 2012), a dataset of United States Army or Marine veterans who were receiving inpatient or outpatient services in the Veterans Affairs (VA) health care system at the time of data collection. As women are underrepresented in the veteran population, women were oversampled at a 1:1 ratio to have sufficient power to conduct appropriate comparisons between women and men. Individuals with a diagnosis of PTSD were oversampled at a 3:1 ratio based on diagnoses in their electronic medical record for two separate VA visits between July 2008 and December 2009. Oversampling based on PTSD diagnosis was conducted during recruitment to create a rich dataset in which group differences could be examined between veterans diagnosed with PTSD and those without PTSD. Although examining group differences was not an aim of the present study, oversampling individuals with PTSD allowed for the examination of a sample with a wider range of total PTSD symptom severity. As additional inclusion criteria, participants were required to have received a mental health evaluation at a VA facility and not be participating in clinical intervention research at the beginning of data collection. In addition, veterans had to have been separated from active duty after serving in support of combat operations in Afghanistan and Iraq following the September 11, 2001, terrorist attacks or completed at least one Reserve or Guard deployment in support of these operations.

### Procedure

Project VALOR investigators (Rosen et al., 2011) identified eligible individuals and mailed letters introducing the opportunity to participate and asking if veterans were interested in further information about the study. Interested individuals completed a phone interview with a doctoral‐level clinician, during which participants provided informed consent and information that could not be obtained from their electronic medical records. Participants then completed an online survey composed of self‐report questionnaires, including those listed in the Measures section. For the present investigation, we used data collected on demographic characteristics; PTSD symptom severity, as measured using the PTSD Checklist for *DSM‐5* (PCL‐5; Weathers, Litz, et al., 2013b); cumulative trauma exposure, as assessed using the Life Events Checklist for *DSM‐5* (LEC‐5; Weathers, Litz, et al., 2013a); depressive symptom severity, as assessed using the nine‐item Patient Health Questionnaire (PHQ‐9; Spitzer et al., 1999); reported alcohol use, via Alcohol Use Disorders Identification Test (AUDIT; Saunders et al., 1993); and prior functioning, as measured using the Inventory of Psychosocial Functioning (IPF; Bovin et al., 2018; Marx et al. 2020), derived from Wave 2 data collection. Later functioning was reevaluated at Wave 4, which took place, on average, 2 years following Wave 2 (*M* = 24.98 months, *SD* = 4.25). All procedures for Project VALOR were approved by the VA Boston Healthcare System Institutional Review Board.

### Measures

#### PTSD symptoms

The PTSD Checklist for *DSM‐5* (PCL‐5; Weathers, Litz, et al., 2013b) is a 20‐item, self‐report questionnaire that is used to measure the presence of *DSM‐5* PTSD symptoms. Although Project VALOR verified PTSD diagnosis using the Structured Clinical Interview for *DSM‐5* (SCID‐5; First et al., 2015) at Wave 2, we used the PCL‐5 in our data analyses, as the PCL‐5 provides symptom‐level severity data. On the PCL‐5, participants rated how much they were bothered by each symptom in the past month, using a 5‐point scale ranging from 0 (*not at all*) to 4 (*extremely*). Total scores range from 0 to 80, with higher scores indicating more severe psychopathology. The PCL‐5 has demonstrated adequate fit with *DSM‐5* symptom clusters (Blevins et al., 2015) and has been validated against the Clinician‐Administered PTSD Scale for *DSM‐5* (CAPS‐5; Weathers, Blake, et al., 2013). For this investigation, we divided the PCL‐5 items into seven factors, consistent with the seven‐factor hybrid model introduced by Armour and colleagues (2015). The seven factors include Anhedonia (Items 12–14), the primary factor of interest; as well as Intrusions (Items 1–5); Avoidance (Items 6–7); Negative Affect (Items 8–11); Externalizing Behaviors (Items 15–16); Anxious Arousal (Items 17–18); and Dysphoric Arousal (Items 19–20). Given that Armour et al. (2015) denote the emotional numbing construct (i.e., diminished interest or participation in significant activities, detachment or estrangement from others, dampened positive affect) as Anhedonia, and the factor structure used in the present study is an important methodological piece, the term “anhedonia” is used from this point forward. In the present sample, internal reliability for the PCL‐5 total score was excellent, Cronbach's α = .96. Cronbach's alpha values for symptom‐specific subscales were .91 for Anhedonia, .92 for Intrusions, .92 for Avoidance, .83 for Negative Affect, .69 for Externalizing Behaviors, .88 for Anxious Arousal, and .72 for Dysphoric Arousal.

#### Psychosocial functioning

The IPF (Bovin et al., 2018; Marx et al. 2020) is an 80‐item, self‐report questionnaire assessing psychosocial functioning in the following domains: romantic relationship with spouse or partner (11 items); family (seven items); work, including home‐based work (21 items); friendships and socializing (eight items); parenting (10 items); education, including distance learning (15 items); and self‐care (eight items). At the start of each domain subscale except for Self‐Care, respondents are asked if that domain applies to them (e.g., parenting may not apply to all participants, as some may not have dependents). In the applicable domains, participants were presented with items (e.g., in the parenting domain, “I had trouble communicating with my children.”) and asked to rate how often that event occurred within the past 30 days, scoring responses on a 7‐point scale ranging from 0 (*never*) to 6 (*always*). Total IPF scores range from 0 to 480, and higher scores indicate higher levels of impairment in psychosocial functioning. In the present sample, Cronbach's alpha for the IPF was .73.

#### Depressive symptoms

The Prime‐MD PHQ‐9 (Spitzer et al., 1999) contains nine items assessing symptoms of depression. Participants were asked to rate symptoms they have experienced over the past 2 weeks (e.g., “feeling tired or having little energy”) using a 4‐point scale ranging from 0 (*not at all*) to 3 (*nearly every day*). Total scores range from 0 to 27, with higher scores indicating higher levels of depressive symptom severity. Based on the *DSM‐5* diagnostic criteria, a probable diagnosis of major depressive disorder is assigned if at least five of the nine items are endorsed with a score of 3 (*nearly every day*). In the present sample, Cronbach's alpha for the PHQ‐9 was .90.

#### Alcohol use

The AUDIT (Saunders et al., 1993) is a 10‐item, self‐report questionnaire that is used to measure potentially problematic alcohol use. The first eight items have five possible responses, with scores ranging from scores of 0 to 4 and customized response anchors for each item. The last two items are measured on a 3‐point scale (0 = *no*; 2 = *yes, but not in the past year*; 4 = *yes, during the past year*). Total scores range from 0 to 40, and higher scores imply an increased risk of alcohol‐related problems. A cutoff score of 8 commonly denotes a possible problematic alcohol use (Saunders et al., 1993). In the present sample, Cronbach's alpha for the AUDIT was .88.

#### Lifetime trauma exposure

The LEC‐5 (Weathers, Litz, et al., 2013a) lists 17 broad trauma categories that may potentially meet *DSM‐5* PTSD Criterion A, or what the *DSM‐5* considers a “traumatic event” (APA, 2013; e.g., fire or explosion, sexual assault, harm done to others). For each type of event, participants report if they have experienced a traumatic event that would fit in that particular category and how they were exposed to the event (i.e., direct experience, witnessing others’ experience, learning of the event happening to a close associate, or being repeatedly exposed to the event as part of their profession). Participants were allowed to endorse multiple exposure types for each trauma category, with each counting as a separate exposure. For the present study, we summed all LEC‐5 responses endorsed at Wave 2 to create a composite trauma exposure score, with higher scores indicating more cumulative trauma exposure. In the present sample, Cronbach's alpha for the LEC‐5 was .80.

### Data analysis

Multiple imputation was used as a method of handling missing data, specifically to retain participants’ data that would have been lost between Waves 2 and 4 due to attrition. Multiple imputation was conducted on all variables used within the analyses described. Five iterations of missing values were imputed, and the reported results were obtained by averaging the parameter estimates across all five iterations.

Given that most IPF subscales, with the exception of Self‐Care, only applied to individuals who endorsed participation in that specific domain (e.g., the Parenting subscale only pertains to those who have parenting responsibilities), independent‐samples *t* tests (for continuous variables) and chi‐square tests (for categorical variables) were conducted for each subscale to examine group differences between participants who endorsed a domain and those who did not. Potential group differences were examined within age, gender (woman, man), race (White, non‐White), ethnicity (Hispanic/Latino, non‐Hispanic/Latino), reported alcohol use, depressive symptom severity, cumulative trauma exposure, total PCL‐5 score, and each of the seven PTSD symptom clusters. Group differences were examined using applicable IPF subscales at Wave 2 (i.e., Romantic Relationship, Family, Friendships, Parenting, Work, Education) given that PCL‐5 scores and all covariates were assessed at Wave 2.

Bivariate correlations were conducted using Wave 2 baseline data. Variables included IPF total score, symptom‐specific scores for the seven PTSD factors (i.e., Intrusions, Avoidance, Negative Affect, Anhedonia, Externalizing Behaviors, Anxious Arousal, Dysphoric Arousal), and PCL‐5 total score. Next, a longitudinal multivariable regression analysis was run with the total IPF score at Wave 4 as the outcome variable and all potentially confounding variables (i.e., age, gender, ethnicity, race, cumulative trauma exposure, depressive symptom severity at Wave 2, reported alcohol use at Wave 2), prior psychosocial functioning (i.e., IPF) at Wave 2, and Wave 2 PCL‐5 total score as predictors. Finally, a multivariable regression analysis was conducted to examine the longitudinal effects of Wave 2 PTSD symptom factors on psychosocial functioning at Wave 4. Covariates included variables derived from Wave 2 data, including demographic characteristics (i.e., age, gender, race, and ethnicity), depressive symptom severity, reported alcohol use, cumulative trauma exposure, and prior psychosocial functioning (i.e., IPF) at Wave 2.

## RESULTS

Table 1 provides sample characteristics derived from Wave 2, including demographic characteristics (i.e., age, gender, race, ethnicity), reported alcohol use, and depressive symptom severity at Wave 2, as well as Wave 2 PCL‐5 total score. Table 1 also includes the number of participants who completed each IPF functioning subscale at Wave 2. To examine possible differences between participants who did and did not endorse different functioning domains as applicable to them, the results of analyses examining group differences in demographic characteristics, covariates, and PCL‐5 data are provided in Supplementary Table S1. In general, veterans who denied participation in Wave 2 functioning domains were older and demonstrated more severe PTSD and depressive symptoms.

**TABLE 1:** Sample demographic characteristics at Wave 2

Variable	*n*	%	*M*	*SD*
Women	825	50.0		
White race	1,036	62.8		
Latino ethnicity	216	13.1		
Age at Wave 2 years			40.91	9.78
Alcohol use (AUDIT)			5.97	6.32
Depressive symptoms (PHQ‐9)			11.72	6.37
Trauma exposure (LEC‐5)			11.71	5.78
PTSD symptoms (PCL‐5 total score)			39.34	18.49
IPF domains^a^				
Romantic relationships	1,090	66.1		
Family	1,511	91.6		
Friendships	1,367	82.9		
Parenting	1,064	64.5		
Work	1,094	66.3		
Education	513	31.1		

*Note*: *N* = 1,649. AUDIT = Alcohol Use Disorders Identification Test; PHQ‐9 = nine‐item Patient Health Questionnaire; LEC‐5 = Life Events Checklist for *DSM*‐*5*; PTSD = posttraumatic stress disorder; PCL‐5 = PTSD Checklist for *DSM‐5*; IPF = Inventory of Psychosocial Functioning.

^a^ Includes the number of participants who completed each IPF functioning subscale at Wave 2.

Bivariate correlations (Table 2) demonstrated that Wave 2 psychosocial functioning (i.e., IPF total score), the Wave 2 PTSD symptom factor scores, and Wave 2 total PCL‐5 score were all significantly correlated with one another, *r*s = .253–.865, *p*s < .001. Anhedonia demonstrated the strongest positive correlation with Wave 2 psychosocial functioning, *r* = .406, compared with the other six PTSD symptom factors: Intrusions, *r* = .293; Avoidance, *r* = .277; Negative Affect, *r* = .356; Externalizing Behaviors, *r* = .361; Anxious Arousal, *r* = .253; Dysphoric Arousal, *r* = .349. However, as mentioned, all correlations were statistically significant.

**TABLE 2:** Bivariate cross‐sectional correlations at Wave 2

Variable	1	2	3	4	5	6	7	8	9
1. Total IPF	–	.293	.277	.356	.406	.361	.253	.349	.395
2. Intrusions		–	.728	.692	.631	.608	.681	.653	.895
3. Avoidance			–	.639	.581	.495	.579	.548	.796
4. Negative affect				–	.712	.655	.555	.594	.865
5. Anhedonia					–	.653	.566	.628	.834
6. Externalizing behaviors						–	.572	.563	.768
7. Anxious arousal							–	.629	.778
8. Dysphoric arousal								–	.781
9. Total PCL‐5									–

*Note*: All correlations were statistically significant at *p* < .001. IPF = Inventory of Psychosocial Functioning; PCL‐5 = Posttraumatic Stress Disorder Checklist for *DSM*‐*5*.

Next, we conducted a longitudinal multivariable regression analysis with the total IPF score at Wave 4 as the outcome variable, and all potentially confounding variables, Wave 2 total IPF score, and Wave 2 total PCL‐5 score as possible predictors (Table 3). Wave 2 psychosocial functioning and total PTSD symptom severity significantly predicted Wave 4 psychosocial functioning. Age and reported alcohol use at Wave 2 also significantly predicted Wave 4 functioning.

**TABLE 3:** Effect of total Wave 2 posttraumatic stress disorder symptom severity on Wave 4 functioning

Variable	*B*	*SE B*	β	*p*
Constant	90.672	9.165		.000
Age at Wave 2	−0.399	0.170	−.042	.020
Gender	−2.103	3.656	−.015	.569
Ethnicity	−0.123	4.838	−.001	.980
Race	−1.917	3.670	−.013	.604
Trauma exposure	0.313	0.270	.026	.246
Alcohol use	−0.690	0.260	−.062	.009
Depressive symptoms	0.446	0.418	.040	.291
Total IPF at Wave 2	0.458	0.024	.472	.000
Total PCL‐5	0.329	0.138	.087	.019

*Note*: *R* ^2^ = .288. IPF = Inventory of Psychosocial Functioning; PCL‐5 = PTSD Checklist for *DSM‐5*.

Finally, we used Wave 2 PTSD symptom factor scores to predict total IPF scores at Wave 4 to examine longitudinal associations between the seven PTSD symptom factors and later psychosocial functioning. In a multivariable longitudinal regression analysis, all Wave 2 symptom factors were simultaneously included with potentially confounding variables and prior psychosocial functioning at Wave 2; these results are presented in Table 4. Similar to the model that included PCL‐5 total score, Wave 2 psychosocial functioning, age, and alcohol use significantly predicted Wave 4 psychosocial functioning. Of the PTSD symptom factors, Anhedonia emerged as the only statistically significant predictor of psychosocial functioning at Wave 4, even when adjusting for prior psychosocial functioning at Wave 2. This suggests that the specific symptoms comprising Anhedonia are driving the association between PTSD symptoms and impairment in psychosocial functioning.

**TABLE 4:** Effect of Wave 2 posttraumatic stress disorder symptom factors on Wave 4 functioning

Variable	*B*	*SE B*	β	*p*
Constant	92.400	9.414		.000
Age at Wave 2	−0.403	0.173	−.056	.021
Gender	−2.409	3.643	−.017	.512
Ethnicity	1.150	4.869	.005	.814
Race	−2.128	3.682	−.015	.566
Trauma exposure	0.326	0.268	.027	.224
Alcohol use	−0.686	0.257	−.061	.008
Depressive symptoms	0.251	0.495	.022	.617
Total IPF at Wave 2	0.447	0.024	.461	.000
Intrusions	−1.036	0.676	−.076	.138
Avoidance	1.286	1.113	.046	.256
Negative affect	0.436	0.629	.027	.490
Anhedonia	2.454	0.906	.123	.011
Externalizing behaviors	−0.044	1.170	−.001	.970
Anxious arousal	0.713	1.078	.025	.512
Dysphoric arousal	−0.376	1.198	−.012	.755

*Note*: *R* ^2^ = .296. IPF = Inventory of Psychosocial Functioning.

## DISCUSSION

This study addressed significant limitations of prior research investigating the impact of PTSD‐related anhedonia on psychosocial functioning across several domains. Data from Project VALOR, a longitudinal registry of United States veterans with and without PTSD, were analyzed and included individuals who completed both Waves 2 and 4 of the study. Longitudinal regression analyses were conducted to investigate how specific clusters of PTSD symptoms at Wave 2 (i.e., intrusions, avoidance, negative affect, anhedonia, externalizing behaviors, anxious arousal, dysphoric arousal) were associated with psychosocial functioning at Wave 4 (i.e., approximately two years later). While simultaneously accounting for prior psychosocial functioning, demographic characteristics, depressive symptoms, alcohol use, and cumulative trauma exposure, PTSD‐related anhedonia was the only statistically significant PTSD factor in its prediction of impairment in later psychosocial functioning above and beyond the contributions of other PTSD symptom clusters. In general, these findings both support and extend conclusions from prior research (Schuman et al., 2019). However, this study expanded upon prior research by addressing important limitations, such as using updated *DSM‐5* diagnostic criteria, isolating specific anhedonia symptoms, employing a longitudinal design, and controlling for important covariates (e.g., depressive symptoms).

As expected, prior psychosocial functioning (i.e., Wave 2 IPF score) significantly predicted later psychosocial functioning (i.e., Wave 4 IPF score). Interestingly, age and reported alcohol use were also robust predictors of later psychosocial functioning. These findings show that older individuals were less likely to endorse participation in almost all functioning domains on the IPF except for parenting; there are several possible reasons for this finding (e.g., older veterans may more often be in retirement). We only found a group difference in reported alcohol use on the IPF Work and Education subscales. However, the contribution of prior alcohol use on later psychosocial functioning is expected given that alcohol use can cause detrimental effects on social relationships, occupational aspirations, and general self‐care.

Given that PTSD is highly comorbid with depression (Rytwinski et al., 2013) and that anhedonia is characteristic of both disorders, preexisting depressive symptoms were included as a covariate in the analyses. We also included other potentially confounding variables. Even with the inclusion of these covariates, anhedonia remained a significant predictor of later impairment in psychosocial functioning. Future research may expand upon this list of covariates or analyze potential moderators that may impact the strength of the association between anhedonia and psychosocial functioning. Other factors may involve the frequency and consistency of participation in treatment, diagnosed medical conditions, the use of medications, or individuals’ current residential and financial situations.

The longitudinal design of this study is also an important feature to note, as it provides evidence that PTSD symptoms precede impairments in psychosocial functioning associated with the disorder. Wave 2 PTSD symptoms strongly predicted functional impairment as measured at Wave 4 even after accounting for functioning deficits that were already present at Wave 2. The finding that anhedonia predicted later functional impairment even when controlling for prior impairment offers strong evidence that PTSD anhedonia symptoms worsen functional impairment over time. These findings highlight the importance of breaking down PTSD symptoms into factors, as the impact of anhedonia symptoms on psychosocial impairment may have been missed by the heterogenous conglomerate of the total PCL‐5 score.

The measure of psychosocial functioning used in the present study (i.e., the IPF) first asks individuals whether they have participated in a specific domain of functioning (e.g., education) and allows nonparticipatory individuals to skip a given subscale if they indicate it does not apply to them. As such, we analyzed potential group differences between the participatory and nonparticipatory groups for each subscale for which this was applicable (i.e., all subscales but Self‐Care). We found that participants who were older, reported more severe depressive symptoms, and reported higher levels of PTSD symptom severity were less likely to endorse participation in functioning domains. Given that the assessment of functional impairment used here, the IPF, excludes participants who do not endorse domain participation to any degree, the results may not be capturing individuals who present with the highest level of functional impairment. Avoidance of or inactivity in particular functioning domains may be influenced by anhedonia. For example, anhedonic individuals may avoid, have little interest in, or have extreme difficulty engaging in specific activities. The advantage of the IPF is its inclusion of a wide variety of functioning domains, thus capturing a larger number of participants who may be excluded from a narrower measure of functioning (e.g., measures that specifically focus on social or occupational functioning). Nonetheless, future research may assess reasons why individuals do not endorse specific functioning domains (e.g., why they are unemployed, why they do not have contact with friends).

PTSD is a heterogenous disorder with numerous variations in symptom presentation, which may be difficult for clinicians to navigate in the treatment process. The current findings present an interesting contribution to the literature such that anhedonia is not traditionally viewed as one of the core features of PTSD. PTSD is traditionally viewed as a fear‐related disorder (Foa, 2011; Foa & Kozak, 1986), and anhedonia and other negative alterations in cognition are not included in the most recent edition of the *International Classification of Diseases* (11th rev.; World Health Organization, 2019). Our findings, along with those reported in previous research, suggest that symptoms of anhedonia may be the most impairing for some individuals. It should be noted, however, that the effect of anhedonia on later psychosocial functioning was relatively small (i.e., β = .123).

The findings presented here may be useful in guiding clinicians to address anhedonia symptoms as most detrimental to psychosocial functioning and overall quality of life. Prior research has shown positive emotions to be important in the use of psychological resources (e.g., attention, knowledge) as well as in the pursuit of incentive‐based goals (e.g., education; Fredrickson, 1998). Therapeutic techniques shown to improve anhedonia include behavioral activation therapy (e.g., Cernasov et al., 2021; Dichter et al., 2009), positive affect treatment (e.g., Craske et al., 2019), and cognitive therapy (e.g., Cernasov et al., 2021). Addressing anhedonia symptoms through the use of empirically validated treatment techniques may improve participation and impairment in several functioning domains, which may improve individuals’ participation in treatment and subsequent treatment outcomes.

In conclusion, PTSD‐related anhedonia symptoms emerged as the strongest predictor of impairment in psychosocial functioning over and beyond the effects of other PTSD symptom factors, in a sample of U.S. Army and Marine veterans. In general, the present findings remain consistent with prior research; however, important limitations of prior research were addressed to strengthen the field's understanding of anhedonia as it relates to posttraumatic stress.

## OPEN PRACTICES STATEMENT

The study reported in this article was not formally preregistered. The data have not been made available on a permanent third‐party archive because the Department of Veterans Affairs does not permit this; requests for the data may be sent via email to the lead author at clmay@uncg.edu .

## Supporting information



Table S1. Group Differences between Endorsed and Non‐Endorsed Functioning Domains
