# Supplementary material for: Posttraumatic stress disorder–related anhedonia as a predictor of psychosocial functional impairment among United States veterans
Source: J Trauma Stress. 2022 Apr 11;35(5):1334–42. doi: 10.1002/jts.22832 (PMC9790620; doi:10.1002/jts.22832)
Supplement: Supplementary file 1 — Table S1. Group Differences between Endorsed and Non‐Endorsed Functioning Domains [file JTS-35-1334-s001.docx]

**Table S1**

*Group Differences between Endorsed and Non-Endorsed Functioning Domains*

|  | Romantic Relationship | | | Family | | |
| --- | --- | --- | --- | --- | --- | --- |
|  | Endorsed  (*n* = 1,090) | Did Not Endorse  (*n* = 559) |  | Endorsed  (*n* = 1,511) | Did Not Endorse  (*n* = 138) |  |
|  | *n* (%) | *n* (%) | χ^2^ | *n* (%) | *n* (%) | χ^2^ |
| Gender (percent Female) | 510 (46.8) | 315 (56.4) | 14.278*** | 756 (50.0) | 69 (50.0) | .179 |
| Race (percent White) | 727 (66.7) | 309 (55.3) | 21.137*** | 957 (63.3) | 78 (56.5) | 2.606 |
| Ethnicity (percent Latino) | 138 (12.7) | 78 (14.0) | .674 | 197 (13.0) | 19 (13.8) | .211 |
|  | *M* (SD) | *M* (SD) | *t* | *M* (SD) | *M* (SD) | *t* |
| Age at Wave 2 | 39.70 (9.42) | 43.27 (10.06) | -6.789*** | 40.57 (9.64) | 44.59 (10.55) | -3.925*** |
| Trauma Exposure | 11.74 (5.93) | 11.65 (5.47) | .277 | 11.69 (5.83) | 11.85 (5.20) | -.262 |
| Alcohol Use | 5.95 (6.22) | 6.01 (6.52) | -.171 | 5.92 (6.23) | 6.49 (7.20) | -.924 |
| Depressive Symptoms | 10.85 (6.22) | 13.42 (6.32) | -7.364*** | 11.48 (6.30) | 14.39 (6.54) | 5.060*** |
| Total PCL-5 Score | 37.22 (18.71) | 43.47 (17.35) | -6.417*** | 38.47 (18.35) | 48.88 (17.21) | -6.126*** |
| Intrusions | 8.86 (5.15) | 10.07 (5.03) | -4.350*** | 9.04 (4.57) | 11.84 (5.10) | -6.018*** |
| Avoidance | 4.22 (2.54) | 4.83 (2.34) | -4.212*** | 4.34 (2.51) | 5.42 (2.06) | -4.534*** |
| Negative Affect | 6.47 (4.31) | 7.72 (4.13) | -5.324*** | 6.73 (10.64) | 8.67 (4.25) | -4.813*** |
| Anhedonia | 5.71 (3.51) | 7.30 (3.33) | -8.287*** | 6.09 (3.49) | 8.00 (3.41) | -5.616*** |
| Externalizing Behaviors | 2.93 (2.06) | 3.34 (2.05) | -3.398*** | 2.99 (2.03) | 3.89 (2.25) | -4.522*** |
| Anxious Arousal | 4.33 (2.54) | 4.80 (2.33) | -3.291** | 4.40 (2.48) | 5.40 (2.30) | -4.326*** |
| Dysphoric Arousal | 4.80 (2.31) | 5.42 (1.99) | -6.327*** | 4.87 (2.24) | 5.65 (2.07) | 3.521*** |

|  | Friendships | | | Parenting | | |
| --- | --- | --- | --- | --- | --- | --- |
|  | Endorsed  (*n* = 1,367) | Did Not Endorse  (*n* = 282) |  | Endorsed  (*n* = 1,064) | Did Not Endorse  (*n* = 585) |  |
|  | *n* (%) | *n* (%) | χ^2^ | *n* (%) | *n* (%) | χ^2^ |
| Gender (percent Female) | 688 (50.3) | 137 (48.6) | .353 | 492 (46.2) | 333 (56.9) | 17.597*** |
| Race (percent White) | 849 (62.1) | 187 (66.3) | 2.313 | 657 (61.7) | 379 (64.8) | 1.875 |
| Ethnicity (percent Latino) | 179 (13.1) | 37 (13.1) | .089 | 148 (13.9) | 68 (11.6) | 2.143 |
|  | *M* (SD) | *M* (SD) | *t* | *M* (SD) | *M* (SD) | *t* |
| Age at Wave 2 | 40.61 (9.72) | 42.33 (9.96) | -2.380* | 41.91 (9.67) | 39.09 (9.72) | 5.182*** |
| Trauma Exposure | 11.75 (5.82) | 11.48 (5.57) | .666 | 11.57 (5.67) | 11.95 (5.97) | -1.141 |
| Alcohol Use | 5.91 (6.05) | 6.26 (7.50) | -.853 | 5.56 (6.06) | 6.71 (6.70) | -3.527*** |
| Depressive Symptoms | 11.11 (6.20) | 14.69 (6.32) | -8.470*** | 11.72 (6.25) | 11.73 (6.58) | -.028 |
| Total PCL-5 Score | 37.37 (17.96) | 48.87 (18.09) | -9.58*** | 39.77 (18.38) | 38.55 (18.69) | 1.270 |
| Intrusions | 8.81 (5.01) | 11.52 (5.18) | -8.121*** | 9.53 (5.13) | 8.81 (5.14) | 2.628** |
| Avoidance | 4.24 (2.48) | 5.36 (2.36) | -6.501*** | 4.52 (2.46) | 4.26 (2.55) | 1.991* |
| Negative Affect | 6.53 (4.20) | 8.67 (4.24) | -7.564*** | 6.84 (4.23) | 6.99 (4.38) | -.651 |
| Anhedonia | 5.81 (3.41) | 8.35 (3.30) | -10.383*** | 6.22 (3.52) | 6.29 (3.55) | -.339 |
| Externalizing Behaviors | 2.87 (1.97) | 4.01 (2.23) | -8.192*** | 3.04 (2.01) | 3.12 (2.15) | -.748 |
| Anxious Arousal | 4.33 (2.49) | 5.27 (2.31) | -5.518*** | 4.60 (2.49) | 4.28 (2.46) | 2.469* |
| Dysphoric Arousal | 4.78 (2.22) | 5.69 (2.13) | -5.509*** | 5.01 (2.23) | 4.80 (2.23) | 1.768 |

|  | Work | | | Education | | |
| --- | --- | --- | --- | --- | --- | --- |
|  | Endorsed  (*n* = 1,094) | Did Not Endorse  (*n* = 555) |  | Endorsed  (*n* = 513) | Did Not Endorse  (*n* = 1,136) |  |
|  | *n* (%) | *n* (%) | χ^2^ | *n* (%) | *n* (%) | χ^2^ |
| Gender (percent Female) | 537 (49.1) | 288 (51.9) | 1.220 | 285 (55.6) | 539 (47.4) | 7.867** |
| Race (percent White) | 687 (62.8) | 349 (62.9) | .793 | 303 (59.1) | 733 (64.5) | 4.998 |
| Ethnicity (percent Latino) | 128 (11.7) | 88 (15.9) | 5.895* | 80 (15.6) | 131 (11.9) | 4.616* |
|  | *M* (SD) | *M* (SD) | *t* | *M* (SD) | *M* (SD) | *t* |
| Age at Wave 2 | 40.06 (9.40) | 42.57 (10.29) | -4.675*** | 39.23 (9.17) | 41.66 (9.96) | -4.315*** |
| Trauma Exposure | 11.63 (5.96) | 11.85 (5.40) | -.669 | 11.52 (6.12) | 11.79 (5.62) | -.835 |
| Alcohol Use | 6.14 (6.27) | 5.63 (6.41) | 1.380 | 5.18 (5.42) | 6.32 (6.66) | -3.027** |
| Depressive Symptoms | 10.49 (5.93) | 5.63 (6.50) | -11.390*** | 10.64 (5.97) | 12.21 (6.48) | -4.450*** |
| Total PCL-5 Score | 35.94 (17.89) | 46.03 (17.84) | -10.623*** | 37.12 (18.26) | 40.34 (18.52) | -3.276** |
| Intrusions | 8.34 (4.94) | 11.12 (5.03) | -9.845*** | 8.78 (5.00) | 9.50 (5.19) | -2.512* |
| Avoidance | 4.03 (2.49) | 5.21 (2.30) | -9.057*** | 4.25 (2.47) | 4.51 (2.50) | -1.905 |
| Negative Affect | 6.26 (4.14) | 8.15 (4.29) | -8.565*** | 6.49 (4.31) | 7.08 (4.26) | -2.412* |
| Anhedonia | 5.74 (3.46) | 7.25 (3.45) | -8.238*** | 5.70 (3.48) | 6.49 (3.52) | -4.119*** |
| Externalizing Behaviors | 2.86 (1.99) | 3.47 (2.14) | -5.581*** | 2.94 (2.03) | 3.12 (2.08) | -1.603 |
| Anxious Arousal | 4.11 (2.49) | 5.22 (2.31) | -8.218*** | 4.14 (2.49) | 4.65 (2.46) | -3.607*** |
| Dysphoric Arousal | 4.60 (2.26) | 5.60 (2.03) | -8.287*** | 4.80 (2.23) | 5.00 (2.23) | -1.415 |

*Note.* The majority of functioning subscales on the IPF provided participants with the opportunity to skip out if the domain did not apply to them. The subscale Self-Care was administered to all participants. A “valid” sample size for each functioning domain is included, given that data were missing for some participants on varying items. Cumulative trauma exposure, PCL-5 total score, and all PCL-5 symptom clusters were measured at Wave 2. Independent samples *t­*-tests and chi-squared tests were conducted to examine group differences.

**p* < .05 ***p* < .01 ****p* < .001
